# Comorbidity of disruptive behavior disorders and intermittent explosive disorder

**DOI:** 10.1186/s13034-020-00330-w

**Published:** 2020-05-28

**Authors:** Karam Radwan, Emil F. Coccaro

**Affiliations:** 1 Section of Child & Adolescent Psychiatry, Department of Psychiatry and Behavioral Neuroscience, Pritzker School of Medicine, University of Chicago, Chicago, IL USA; 2 Clinical Neuroscience and Psychotherapeutics Research Unit, Department of Psychiatry and Behavioral Health, The Ohio State University Wexner Medical Center, 1670 Upham Drive, Columbus, OH USA

**Keywords:** Attention deficit/hyperactivity disorder, Conduct disorder, Oppositional defiant disorder, Aggression

## Abstract

**Background:**

Aggressive behavior in children and adolescents may be accounted for by several disruptive behavioral disorders (DBD) including attention-deficit/hyperactive (ADHD), conduct (CD), and oppositional defiant (ODD), disorders and intermittent explosive disorder (IED). The comorbidity among the DBDs is well known, but not its comorbidity with IED.

**Method:**

We reanalyzed data from the National Comorbidity Studies (adolescents and adults), and from a large clinical research adult sample, to estimate the comorbidity of IED with each of the DBDs and to explore correlates of these comorbidities.

**Results:**

The rate of current comorbidity between IED and the DBDs ranged from 10 to 19%, in adolescents (5–14% in adults) with odds ratios of about five. The onset of ADHD typically appeared before onset of IED while onset ODD and CD more typically appeared before that of IED in adolescents and about equally before or after IED in adults but IED persisted outside the duration window in many (ADHD) or most (ODD, CD) cases. Measures of impulsive aggression severity were highest in those with IED+DBD but relatively low in those with DBD alone while measures of DBD severity were highest in those with DBD alone and in those with IED+DBD.

**Conclusion:**

Despite the comorbidity of IED with the DBDs, IED can be separated from the DBDs over time and in terms of severity measures of IED and of DBD. Overall, impulsive aggression varies with IED while DBD behaviors vary with DBD. Based on this, clinicians should consider IED in their differential in the workup of impulsively aggressive children and adolescents.

## Background

A critical issue in the field of psychiatry is how to best describe and understand the nature of impulsive aggression in children and adolescents. It is well documented that individuals with early-onset disruptive behavior, including attention-deficit/hyperactive (ADHD), conduct (CD), and oppositional defiant (ODD), disorders are at high risk for later adverse psychosocial outcomes (e.g., school dropout, criminality, substance abuse, reduced social skills, and mental health problems) [1]. Not surprisingly, disruptive behavior disorders (DBD) are common in early life and have reported lifetime rates of 8.1% for ADHD, 12.6% for ODD, and 6.8% for CD [2]. While much less studied in children and adolescents, Intermittent Explosive Disorder (IED), a disorder of recurrent, problematic, impulsive aggression, is also common in young individuals and has a reported lifetime prevalence of 7.8% [3]. Despite the fact that these four disorders share overlapping behaviors, there is no published data examining the relationship between the DBDs and IED.

The validity of IED in adults is now supported by studies showing that IED: (a) can be diagnosed reliably [4], (b) is relatively stable over time [5], (c) is taxonic rather than dimensional in nature [6], (d) runs in families [7], (e) can be separated from other comorbid disorders on a number of relevant variables [8–11] and, (f) correlates with biomarkers of aggression and impulsivity [12]. Despite this, clinicians and researchers working with children and adolescents largely focus on DBDs in the context of anger, impulsivity, and aggression. Making matters more complex, a new disorder in DSM-5, codified as disruptive mood dysregulation disorder (DMDD; [13]) also highlights anger and aggression, though DMDD is primarily conceptualized as a mood disorder. The primary difference between DMDD and IED is that the former represents a severe form of mood disorder in which anger is present most of time occurring before the age of ten while the latter describes individuals in whom aggressive outbursts are frequent but episodic and in whom anger is not present most of the time between outbursts. While not perfectly aligned with children and adolescents, studies in adults suggest that IED is comorbid with DMDD in less than 10% of cases [14] indicting that the two may well be clinically separable.

Clinically, IED and the DBDs may be compared and contrasted in the following ways: (a) ADHD and IED share high levels of impulsive behavior but those with IED manifest serious aggression toward others, which is not characteristic of those with ADHD; in addition those with IED do not experience problems with sustaining attention as seen in ADHD; (b) CD and IED share history of aggressive behavior but this behavior tends to be anger-based/impulsive in IED but predatory/premeditated in CD; and (c) ODD and IED share history of temper tantrums but these are more frequent, and accompanied by more severe aggressive outbursts, in those with IED.

In this paper, we study the comorbidity of IED and DBD based on available empirical data from two large community surveys and from a relatively large clinical research data set. Disruptive Mood Dysregulation Disorder (DMDD) was not included in this study because of none of the data sets collected the data needed to make this diagnosis. We examined several aspects of comorbidity in IED, as well as the relative ages of onset of IED and DBDs. Since reliability is highest when considering concurrent diagnoses, we focused on those conditions present within the last 12 months for most analyses. Additionally, we also examined the quantitative nature of aggression as a function of IED and comorbid DBDs in the two community samples. In the Clinical Research sample we were able to examine the quantitative nature of DBD behavior scores as a function of IED and comorbid DBD. We hypothesized that while current IED would display an increased rate of comorbid DBD disorders, individuals with IED would display: (a) no more overall current comorbidity than those with DBD disorders, (b) ages of onset of IED precede that of each comorbid DBD disorder, (c) similarly elevated aggression scores in those with IED only, those with IED and each DBD, those with DBD only, and (d) elevated DBD scores only in those with DBD and with IED and DBD.

## Methods

This study analyzed data from three sources. First, the National Comorbidity Survey-Adolescent Supplement (NCS-AS; [15]); second, the National Comorbidity Survey -Replication of adults (NCS-R; [16]) and, third, an adult clinical research sample engaged in research studies approved by the University of Chicago Institutional Review Board. The primary data source for this work was the NCS-AS with data from the NCS-R and the clinical research sample serving as a comparison between adolescents and adults. The two NCS data sets are publically available (NCS-AS: http://dx.doi.org/10.3886/ICPSR28581 and NCS-R: http://www.icpsr.umich.edu/icpsrweb/ICPSR/studies/20240).

### Study samples

#### Community samples

The NCS samples constitute surveys of mental disorders in the United States of America. The NCS-AS is made up of 10,148 adolescents of both sexes (mean ± SD age: 15.8 ± 1.5); the NCS-R involved 9282 adults of both sexes (mean ± SD age: 44.7 ± 17.5 years). Details regarding methods of data collection have been published [15, 16].

#### Clinical research sample

The Clinical Research sample contained 1644 adults (mean ± SD: 33.3 ± 9.9 years) of both sexes who had completed at least one study in which research diagnostic/personality trait assessments were completed. Individuals were recruited from the community using public service announcements seeking participants for the various studies. Details regarding the clinical research sample have been published [17].

### Diagnostic assessments

The NCS surveys were designed to yield psychiatric diagnoses according to the DSM-IV [18]. However, raw data in the NCS-AS/NCS-R data bases allowed diagnoses to be updated to DSM-5 [19]. For the IED diagnosis, participants reported at least three “anger attacks” in any given year (Criteria A_2_) and met the remainder of the DSM-5 criteria for IED. Although DSM-5 also allows frequent, but low intensity “anger attacks” (Criteria A_1_), the NCS-AS/NCS-R surveys did not record data related to this type of “anger attack”. Psychiatric diagnoses in the Clinical Research Sample were made using DSM-5 criteria (Criteria A_1_ and A_2_) as previously described [17]. Study participants in this sample with any psychiatric diagnosis (n = 1189), 58% (n = 690) reported a history of formal psychiatric evaluation and/or treatment in 58% (n = 690) of cases; an additional 14% (n = 166) of cases reported a history of behavioral disturbance during which the participant or others thought the participant should have sought mental health evaluation/treatment but did not. The NCS-AS study included data from interviews with the adolescents and from questionnaires about the adolescent by their parents in most (68%), but not all, cases; such informant data was not collected for NCS-R and Clinical Research samples.

### Dimensional variables relevant to IED

Both NCS-AS/NCS-R surveys included six [6] questions very similar to those from established assessments of aggression (e.g., “I have temper tantrums” compared with “I have trouble controlling my temper” from the Buss-Perry Aggression Questionnaire: BPAQ [20] and impulsivity (e.g., “Giving into urges gets me into trouble” compared with “Do you often get into a jam because you do things without thinking?” from the impulsivity scale of the Eysenck Personality Questionnaire [21] which enabled the creation of a variable for impulsive aggression. The scoring for the two surveys differed because the six NCS-AS items had four anchor points (0, 1, 2, or 3) and the six NCS-R items had two anchor points (0 or 3). The Clinical Research data contained aggression scores from the life history of aggression (LHA; [22]) and the verbal and physical assault scores from the Buss-Perry Aggression Questionnaire (BPAQ; [20]), assessments. Psychometric properties for LHA Aggression (e.g., α = 0.88) and for BPAQ Aggression (e.g., α = 0.85 for Physical, and α = 0.73 for Verbal, Assault) are good to excellent.

### Dimensional variables relevant to DBD

The NCS-AS and NCS-R surveys did not include dimensional variables relevant to the severity of DBD disorders. While symptom counts for each DBD could be calculated, the structure of the interviews did not allow for an assessment of all DBD criteria in all subjects and, thus, could not be used. While this was also true for the Clinical Research group, data from the Wender-Utah Rating Scale (WURS [23]), a Likert-scaled questionnaire assessing current and lifetime DBD (and other behaviors) were available in a sizable subset of the Clinical Research study participants (n = 713). The WURS contains twenty items that assess current severity of ADHD (separate scores for Hyperactivity-Impulsivity and Inattention), ODD, and CD, behaviors.

### Statistical analysis

Statistical procedures included binary logistic regression for adjusted odds ratios, analysis of covariance (ANCOVA), and paired t-tests, as appropriate. All reported data was adjusted for age, sex, ethnicity, and education (level for parent for NCS-AS; level for subject for NCS-R) or Hollingshead Socio-Economic Status score (clinical research group). A two-tailed alpha of 0.05 was used to denote statistical significance for all analyses with Bonferroni-correction as appropriate. The first set of analyses involved examined the number of current disorders for each sample. This was followed by an examination of the rates (percentages) and risk (odds ratio) for overall comorbidity (e.g., comorbidity of a disorder with all other disorders). Next, we examined the rates and comorbidity risk for each DBD disorder as a function of IED taken separately as well as examining the comorbidity risk for all disorders in the same statistical model to determine the true comorbid nature of IED. The second set of analyses examined the age (and relative sequence) of onset for IED and each DBD disorder to determine the temporal nature of IED comorbidity. The third set of analyses examined mean aggression scores as a function of comorbidity. For example, subjects in each sample were divided into those with no life history of any disorder, those with a Non-IED/DBD disorder, those with a DBD disorder (e.g., ADHD, ODD, CD), those with IED, and those with both IED and DBD. This was performed to determine if aggression scores were higher in IED compared with those with DBD, and compared with both IED and a DBD (e.g., IED + ADHD). Composite Aggression scores were created for the Clinical Research group by taking the mean z scores for LHA and BDHI scores.

## Results

### Characteristics of NCS-AS/NCS-R participants

Adolescents in the NCS-AS survey were largely self-described as white (White: 74.6%, African–American: 19.3%, other ethnicity: 6.1%) and of nearly equal proportion male (48.9%) and female (51.1%). Nearly a third of the parents of the adolescents had no more than a high school education (32.7%) while a quarter (25.2%) had a partial college experience and a third (33.1%) had a college degree or higher. Adults in the NCS-R survey were largely self-described as white (White: 81.7%, African–American: 13.1%, other ethnicity: 5.1%), and male (45.5%) and female (55.5%). About two-fifths (40.5%) of the NCS-R adults had no more than a high school education while nearly a third (29.4%) had a partial college experience and nearly a third (30.1%) had a college degree or higher.

### Characteristics of the clinical research sample participants

Unlike the two NCS samples, only half the Clinical Research group were mostly self-described as white (White: 54.3%; African–American: 33.7%, other ethnicity: 12.1%). Slightly more of than half the sample were male (56.3% vs. 43.6% female). About a third (33.2%) of these study participants had no more than a high school degree while nearly a third (28.8%) had a partial college experience and a third (33.0%) had a college degree or higher.

### Frequency of current IED and current DBD in the samples (Table 1)

Table 1 lists the frequency of current IED and current DBD in both the adolescent community and adult community sample. Current IED was present in 4.7% of all cases in the adolescent community sample and 2.6% in the adult community sample. Current DBD of any kind was present in 8.6% of all cases in the adolescent community sample with ODD at 4.1%, followed by CD at 3.3%, and ADHD at 2.5%. In contrast, only 2.6% of all study participants in the adult community sample had a current DBD with ADHD at 2.0%, followed by ODD at 0.3% and CD at 0.4%.

**Table 1:** Frequencies of IED and DBD in the three samples

Disorder	NCS-AS reanalysis adolescents (N = 10,148)	NCS-R reanalysis adults (N = 9282)	Clinical research analysis adults (N = 1644)
IED: Current life	6.4% (n = 651) 8.9% (n = 899)	2.6% (n = 238) 4.0% (n = 368)	35.8% (n = 588) 43.2% (n = 709)
ADHD: Current life	2.5% (n = 249) 4.3% (n = 432)	2.0% (n = 190) 3.9% (n = 365)	2.6% (n = 43) 6.9% (n = 113)
ODD: Current life	4.3% (n = 435) 10.3% (n = 1047)	0.6% (n = 55) 4.9% (n = 453)	1.0% (n = 17) 12.3% (n = 201)
CD: Current life	3.3% (n = 335) 5.8% (n = 586)	0.4% (n = 33) 4.4% (n = 405)	1.0% (n = 16) 13.3% (n = 219)

### Overall comorbidity for IED and DBD Disorders

Table 2 displays the mean (± SD) number of all current comorbid disorders with IED and DBD, respectively, in the three study samples. The number of all current disorders comorbid with current IED was statistically similar to that for each DBD disorder for the adolescent community. This was not true for the adult community sample, where number of current disorders comorbid with current IED was significantly lower than that for each of the DBD disorders. Despite this, the odds ratio for overall current comorbidity for current IED was statistically similar to that for each of the DBD disorders (Table 3).

**Table 2:** Mean (± SD) number of current comorbid disorders in participants with IED or DBD

Disorder	NCS-AS Reanalysis Mean # (± SD) of Dx	NCS-R Reanalysis Mean # (± SD) of Dx	Clinical research analysis Mean # (± SD) of Dx
Intermittent explosive disorder	1.43 ± 1.26^a^	1.42 ± 1.52^b^	0.67 ± 0.88^c^
Attention Deficit/hyperactivity disorder	1.81 ± 1.40	2.05 ± 1.69	1.79 ± 1.14
Oppositional defiant disorder	1.48 ± 1.28	2.87 ± 1.90	1.76 ± 1.09
Conduct disorder	1.60 ± 1.37	2.79 ± 2.10	1.31 ± 0.79

^a^T-test: IED < ADHD (t_726_ = 3.71, p < 0.001), IED = ODD (t_912_ = 0.55, p = 0.551), IED = CD (t_812_ = 1.83, p = 0.068)

^b^T-test: IED < ADHD (t_426_ = 4.05, p < 0.001), IED < ODD (t_291_ = 6.07, p < 0.001), IED < CD (t_269_ = 4.61, p < 0.001)

^c^T-test: IED < ADHD (t_629_ = 7.88, p < 0.001), IED < ODD (t_603_ = 5.00, p < 0.001), IED < CD (t_602_ = 2.88, p < 0.005)

**Table 3:** Overall Comorbidity of Current IED Compared with that of Current DBD Disorders

With vs. without disorder	NCS-AS reanalysis OR (95% CI) [% Dx vs. % Other-Dx]	NCS-R reanalysis OR (95% CI) [% Dx vs. % Other-Dx]	Clinical research analysis OR (95% CI) [% Dx vs. % Other Dx]
IED vs. all other Dx	5.68 (4.61–7.04) [73.7% vs. 33.6%]	5.68 (4.31–7.52) [66.4% vs. 23.8%]	2.67 (2.11–3.37) [39.5% vs. 18.8%]
ADHD vs. all other Dx	8.00 (5.81–10.99) [79.9% vs. 35.1%]	11.12 (7.64–16.18) [81.6% vs. 18.4%]	11.90 (4.17–33.33) [90.7% vs. 46.8%]
ODD vs. all other Dx	6.95 (5.58–8.65) [77.6% vs. 22.4%]	22.29 (8.82–56.30) [90.9% vs. 9.1%]	N/A [100.0% vs. 47.6%]
CD vs. all other Dx	6.17 (4.76–8.00) [76.4% vs. 34.5%]	35.72 (8.49–150.27) [93.9% vs. 6.1%]	16.13 (2.10–125.00) [93.8% vs. 47.7%]

### Specific current comorbidity of IED with DBD disorders (Table 4)

Across the two community samples, the most frequent current DBD disorder comorbid with IED varied with the sample. In the adolescent community sample, ODD was more frequent than CD and ADHD in cases of IED + DBD comorbidity. This changed in the adult community sample so that ADHD was more frequent than CD and then ODD. That said, the odds ratios for these comparisons did not differ statistically except for CD. In the clinical research sample ADHD was the most prevalent comorbid diagnosis with IED followed by ODD and CD; the odds ratios for these comparison did not differ from each other and did not differ from the community sample. Comorbidity as a function of biological sex did not differ in any of the samples.

**Table 4:** Odds ratios and frequencies of current DBD co-morbid with current IED

Current IED vs. Current DBD	NCS-AS reanalysis OR (95% CI) [% Dx in IED vs. % in non-IED]	NCS-R reanalysis OR (95% CI) [% Dx in IED vs. % in non-IED]	Clinical research analysis OR (95% CI) [% Dx in IED vs. % in non-IED]
ADHD	5.15 (3.70–7.19) [10.0% vs. 2.1%]	5.36 (3.44–8.34) [14.3% vs. 1.7%]	3.25 (1.71–16.13) [4.6% vs. 1.5%]
ODD	5.40 (4.20–6.94) [19.0% vs. 4.1%]	5.51 (2.61–11.65) [5.0% vs. 0.5%]	N/A [2.9% vs. 0.0%]
CD	4.48 (3.31–6.06) [12.3% vs. 2.9%]	13.71 (6.48–29.04) [5.9% vs. 0.2%]	27.33 (3.57–209.51) [2.6% vs. 0.1%]

### Current comorbidity in the context of IED and DBD disorders (Table 5)

Given the substantial comorbidity of each DBD with IED we placed all current DBD disorders into the same logistic regression model (one model per sample) to determine which DBD disorders had significantly elevated odds ratios for IED comorbidity considering the comorbidity among all DBD disorders. Results across the three samples were consistent with odds ratios of about two for ADHD and ODD and from about two to greater than twenty-five for CD.

**Table 5:** Comorbidity of current IED in the context of all current disorders^a^

Current comorbid DBD	NCS-AS reanalysis^b^ OR (95% CI)	NCS-R reanalysis^b^ OR (95% CI)	Clinical research analysis^b^ OR (95% CI)
ADHD	1.98 (1.37–2.87)***	2.26 (1.39–3.68)***	2.25 (1.13–4.46)*
ODD	2.61 (1.95–3.51)***	1.33 (0.59–3.02)	N/A
CD	2.22 (1.59–3.12)***	5.15 (2.28–11.63)***	29.41 (3.86–250.00)***
Overall model statistics	X^2^ (df = 15) = 390.10 p < 0.001	X^2^ (df = 17) = 327.54 p < 0.001	X^2^ (df = 12) = 188.39 p < 0.001

^a^Current disorders include: any bipolar, depressive, anxiety, substance use, post-traumatic stress; any eating disorder and all of the current DBD disorders (current bipolar and substance use did not apply to the clinical research analysis)

^b^Each column represents the odds ratios from a separate binary logistical regression, adjusted for demographic covariates, for each analysis group. Asterisks represent the alpha level for each DBD in the model (* p < 0.05, ** p ≤ 0.01, *** p ≤ 0.001)

### Age of onset of IED and DBD (Table 6)

Considering current and past disorders, the age of onset for ADHD in the NCS-AS sample was significantly earlier than that for IED while the age of onset for ODD and CD were significantly later than IED. The NCS-R sample replicated the earlier age of onset for ADHD but found that onset of ODD was earlier that IED while that for CD was similar to that for IED. The clinical research sample was similar to the NCS-R sample except that each DBD had earlier ages of onset than IED.

**Table 6:** Mean age of onset (± SD) for lifetime IED with lifetime comorbid DBD disorders

DBD disorder	NCS-AS reanalysis^a^		NCS-R reanalysis^a^		Clinical research analysis^a^	
	IED Age of onset	Comorbid DBD Age of onset	IED Age of onset	Comorbid DBD Age of onset	IED Age of onset	Comorbid DBD Age of onset
ADHD	9.4 ± 3.3	6.9 ± 2.4 (t_105_ = 6.98, p < 0.001)	11.6 ± 6.4	6.5 ± 1.8 (t_62_ = 6.28, p < 0.001)	12.5 ± 5.7	6.3 ± 1.8 (t_108_ = 10.95, p < 0.001)
ODD	9.8 ± 3.2 (t_243_ = 3.09, p < 0.001)	10.3 ± 3.0	11.6 ± 4.3	10.3 ± 3.5 (t_80_ = 2.57, p = 0.012)	12.7 ± 5.6	11.2 ± 3.3 (t_170_ = 3.36, p = 0.001)
CD	9.9 ± 3.4 (t_152_ = 8.56, p < 0.001)	12.6 ± 2.4	11.7 ± 4.5	11.6 ± 3.3	12.3 ± 5.4	10.7 ± 1.4 (t_185_ = 3.90, p < 0.001)

^a^Difference between IED vs. DBD by paired T-test

### Temporal order of IED and DBD (Table 7)

Next, we examined the temporal overlap of IED with the DBDs (i.e., age of onset vs. age when the disorder remitted) in the NCS-AS and NCS-R samples (similar data was not available in the third sample). For the NCS-AS adolescent sample, the temporal period of IED was fully contained within that of DBD in half of cases with ADHD (54.3%), a third with ODD (32.8%), and a fifth with CD (22.0%), suggesting that nearly half of adolescents with comorbid ADHD, and most with comorbid ODD or CD, have a period of active IED in the absence of active DBD. Even in the reverse situation, in whom the DBD appeared first (20.3% to 69.8% of cases), the period of IED extended beyond that of the DBD in a third of cases (i.e., 35.6% for ADHD, 33.8% for ODD, and 35.5% for CD) and, again, present in the absence of active DBD indicating that IED can be separated from the DBDs over time in many cases. Severity of IED, as assessed by greatest number IED episodes in any year, did not differ as a function of the temporal order of IED/DBD. Results from the NCS-R adult sample were similar. The temporal period of IED was fully contained within that of DBD in less than two-fifths of cases with ADHD (38.1%) and less than one-sixth in ODD (14.8%) or CD (13.5%), indicating that most of those with comorbid DBD have active IED in the absence of an active DBD. For those in whom the DBD appeared first (40.0% to 68.3% of cases), the temporal period of IED extended beyond that of DBD in about half of cases with ADHD (51.2%) and about one-fifth with ODD (19.0%) or CD (21.4%). Similar to the NCS-AS sample, severity of IED did not differ as a function of the relative temporal order of IED/DBD.

**Table 7:** Temporal association of disorder onset in lifetime IED with lifetime comorbid DBD diagnoses

DBD disorder	NCS-AS reanalysis^a^		NCS-R reanalysis^a^		Clinical research analysis^a^	
	IED First	DBD First	IED First	DBD First	IED First	DBD First
ADHD	18.3%	70.0% (z = 14.52, p < 0.001)	23.3%	68.3% (z = 8.30, p < 0.001)	9.1%	83.6% (z = 26.88, p < 0.001)
ODD	47.7% (z = 5.76, p < 0.001)	31.1%	32.1%	50.0% (z = 3.33, p < 0.001)	36.0%	48.0% (z = 3.19, p < 0.001)
CD	67.6% (z = 12.92, p < 0.001)	20.0%	44.3%	40.0%	40.3%	54.3% (z = 3.82, p < 0.001)

^a^Difference between IED First vs. DBD First by test of proportions (z)

### Magnitude of aggression/impulsivity scores as a function of comorbidity (Tables 8, 9)

Finally, comparing levels of aggression as a function of lifetime DBD comorbidity found that composite aggression scores increased in a stepwise fashion going from those with no lifetime disorder to those with a Non-IED/Non-DBD, to those with a DBD, to those with IED, to those with IED+DBD. Composite Aggression scores differed significantly across all groups with IED + DBD study participants having the highest Composite Aggression scores.

**Table 8:** PEA aggression scores (Z-transformed) as a function of lifetime DBD disorder and comorbidity with lifetime IED in adolescent community sample [ANCOVA: Marginal Means (± SEM) in NCS-AS Reanalysis]

Comorbid DBD (adolescent sample)	No disorder	Other disorder	Specific DBD	IED	IED+Specific DBD	ANCOVA
ADHD	− 0.26 ± 0.02	0.20 ± 0.02	0.70 ± 0.05	0.76 ± 0.03	1.15 ± 0.09^*^	F[4,10,139] = 397.03 p < 0.001
ODD	− 0.26 ± 0.02	0.11 ± 0.02	0.74 ± 0.03	0.70 ± 0.04	1.07 ± 0.06^*^	F[4,10139] = 467.40 p < 0.001
CD	− 0.26 ± 0.02	0.18 ± 0.02	0.68 ± 0.05	0.72 ± 0.04	1.19 ± 0.08^*^	F[4,10139] = 407.45 p < 0.001

See text for details on calculation of scores. PEA aggression scores were Z-transformed for ease of comparing data in

Tables 8 and 9. All post hoc comparisons significant (p < 0.001) except for comparisons for IED vs. Specific DBD which were not statistically significant (all p > 0.10)

**Table 9:** PEA aggression scores (Z-transformed) as a function of lifetime DBD disorder and comorbidity with lifetime IED in adult community sample [ANCOVA: marginal means (± SEM) in NCS-AS reanalysis]

Comorbid DBD (adult sample)	No disorder	Other disorder	Specific DBD (No IED)	IED (No DBD)	IED+specific DBD	ANCOVA
ADHD	− 0.36 ± 0.02	0.11 ± 0.02	0.65 ± 0.05	1.02 ± 0.05	1.55 ± 0.11	F[4,6646] = 294.10 p < 0.001
ODD	− 0.36 ± 0.02	0.09 ± 0.02	0.76 ± 0.05	1.01 ± 0.05	1.47 ± 0.10	F[4.6646] = 320.78 p < 0.001
CD	− 0.36 ± 0.02	0.11 ± 0.02	0.63 ± 0.05	0.99 ± 0.05	1.60 ± 0.10	F[4,6646] = 296.62 p < 0.001

All post hoc comparisons between the five groupings were significant (p < 0.001)

### Magnitude of aggression and DBD severity scores as a function of comorbidity (Fig. 1)

**Fig. 1:**
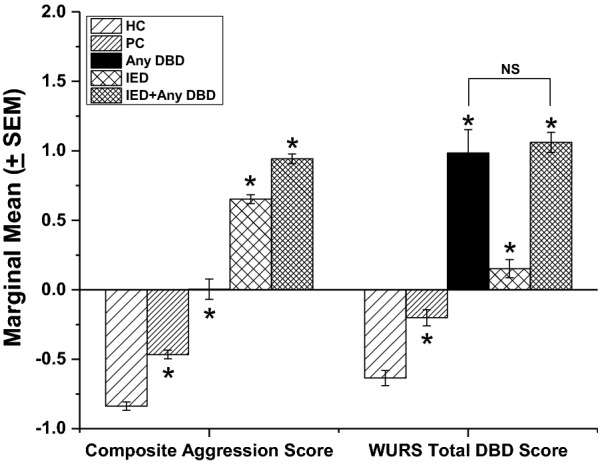
Marginal means (± SEM), after ANCOVA, for composite aggression score and WURS Total DBD score in healthy (HC) and psychiatric (PC) controls, any DBD, IED, and those with IED+DBD. ANCOVA for composite aggression scores: F[4,1548] = 518.71, p < 0.001; all post hoc comparisons were significant (p < 0.001). ANCOVA for WURS Total DBD scores: F[4,1548] = 96.77, p < 0.001; all post hoc comparisons were significant (p < 0.001) except for Any DBD vs. IED+Any DBD (p = 0.513). Asterisks indicate statistically significant differences between all other groups

Finally, we compared severity levels of composite aggression, and lifetime DBD behaviors using WURS scores, as a function of comorbidity in the Clinical Research sample. This analysis revealed that those with IED + DBD had the highest aggression score followed, in a stepwise fashion, by those in the IED, DBD, Psychiatric Control and Healthy Control groups. In contrast, Total DBD WURS scores were highest, and statistically similar, for the DBD and IED+DBD (Fig. 1). Those with IED alone had lower Total DBD WURS scores than either DBD or IED + DBD group but a higher DBD WURS score than either Psychiatric, or Healthy, Controls. Since Composite Aggression scores were correlated with Total WURS DBD scores (r = 0.54, p < 0.001), we repeated the DBD WURS analysis using Composite Aggression scores as a covariate and found the same results. Results from a similar analysis for ADHD-Hyperactivity, ADHD-Inattention, CD, and ODD were the same for each DBD severity variable.

## Discussion

Reanalysis of data from three different samples strongly suggests, with few exceptions, that: (a) comorbidity of current IED with current DBDs is similar to (or less than) current comorbidity of DBD disorders with other disorders; (b) taking all examined disorders simultaneously, current IED is significantly comorbid with each DBD with an odds ratio of about two; and, (c) mean aggression scores are highest among those with IED+DBD followed by those with IED alone, DBD alone, Psychiatric Controls, and Healthy Controls suggesting that the comorbidity of a DBD with the presence of IED is associated with an even greater severity of aggressive behavior than of IED alone. Notably, the reverse was not observed. When examining severity of DBD across the groups we observed that elevated DBD scores are characteristic of only those with DBD regardless of whether they also had IED. This suggests that DBD symptoms are unlikely to explain the aggressiveness of those with IED.

Examination of the temporal relationship of the disorders is important in understanding links between IED and DBD. The results were clear for ADHD, but less so for ODD and CD. In all three samples, the reported onset of ADHD was several years before that for IED, suggesting that the presence of ADHD increases the risk for developing IED at a later time. When examining both current and past diagnoses, the presence of IED persisted beyond the time of active ADHD in nearly half of adolescent cases (45.7%) and in the vast majority of adult cases (88.6%). Thus, despite the earlier onset of ADHD, the two disorders can be distinguished over time. Finally, while the risk of IED in those with ADHD is greater than in those without ADHD, only a quarter of adolescents (24.5%) with lifetime ADHD were comorbid for lifetime IED, indicating that lifetime comorbidity with IED does not account for most cases of ADHD. The reverse was also true with about an eighth of adolescents with lifetime IED (12.7%) having lifetime ADHD.

For ODD and CD, examination of the NCS-AS sample revealed that IED manifests itself before ODD in a plurality of cases (45.9%) and before CD in the majority of cases (69.3%). This was not true in the NCS-R and Clinical Research adult samples where the proportions of IED occurring first were lower, or about the same, as that occurring after ODD or CD. Given that the individuals in the NCS-AS sample were adolescents at time of study, and that reported history of psychopathology would be less affected by retrospective assessment in this sample (compared with the adult samples), one may give more weight to the results from the NCS-AS adolescent sample and suggest that IED manifests earlier than ODD and CD in more cases than not. Similar to ADHD, active IED was present when ODD (66.2%) or CD (78.0%) was not active, indicating that IED and ODD or CD can also be distinguished from IED over time. Finally, while the presence of IED may increase the risk of developing ODD or CD, less than a third with lifetime IED had lifetime ODD (29.2%) and less than a fifth had lifetime CD (18.3% %), indicating that lifetime comorbidity with ODD or CD does not account for most cases of IED.

Taken together, these data support the hypothesis that IED is a discrete disorder in adolescents in the same manner that DBDs are considered discrete disorders. That is, IED is not excessively comorbid with other current disorders to render it better explained by the presence of other another disorder or psychopathology, it occurs relatively early in life, and elevated aggression scores are characteristic of IED with or without the presence of a comorbid DBD disorder. Since aggressive behaviors in childhood and adolescence are associated with multiple undesirable outcomes, including juvenile delinquency, academic failure, and substance abuse, identifying IED, and making a proper diagnosis early in childhood, might provide a developmental opportunity to intervene and mitigate risk factors associated with aggression [24].

This study has strengths and limitations. First among strengths, these results are based on a reanalysis of two large population-based community data sets and one relatively large clinical research data set. Second, diagnoses were updated to those of DSM-5, though only the A_2_ criteria for IED were applied (because questions relevant to the A_1_ criteria were not included in the survey instruments used at the time). That said the clinical research data set assessed IED by both A_1_ and A_2_ criteria and the results of these analyses rendered similar results as those with the community survey data set. Third, we were able to assess a variable for aggression in all samples and a variable for DBD severity for the clinical research sample, and found similar results.

This report differs from a previous report using the NCS-AS data set. First, the McLaughlin et al. [3] report did not include all subjects who entered the NCS-AS study. This report included only participants 17 or younger and only participants with data from parent informants. Instead, we included 598 participants aged 18 and we included 3067 participants without data from paired informants. That said, our general results regarding prevalence of IED and the DBDs are similar suggesting that adding these data did not materially affect the reported findings.

This examination has limitations as well. First, the community sample data set was collected in the early 2000s and there may have been changes in the community-based epidemiology of IED and the other disorders examined. Unfortunately, we are not aware of another relevant community data targeted community survey to take place. Second, self-reported data is subject to retrospective bias and the presence or absence of disorders and the timing of onset of disorders could be affected by this factor. This is why we largely limited this analysis to examining current/past year disorders. Third, while two-thirds of the NCS-AS sample had parent informants, no informant interviews were conducted in the other two data sets. While desirable, informant interviews were not possible due to the expense this would have entailed. Fourth, our data regarding aggression severity in the community sample was derived from a group of personality items and not from full assessments of impulsive aggression as in the clinical research sample. That said, these items were drawn from established measures and results were consistent with that in the clinical research sample.

The present study adds to the growing body of literature on the comorbidities of IED. We emphasize that child and adolescent psychiatrists should think about IED in the deferential of DBD because early recognition of IED may help guide the treatment of aggressive behavior in such individuals. It is important to highlight that the presence of IED does not appear to alter the severity of DBD scores.

## Conclusions

The presence of IED as a comorbid disorder appears to be associated with significant additional morbidity and complicates the diagnosis, treatment, and prognosis of DBDs. Multidisciplinary research should focus on investigating underlying mechanisms related to aggression in IED and comorbid DBD, as well as the utility of various treatment modalities.

## Data Availability

The datasets used and/or analyzed during the current study are available from the corresponding author on reasonable request.
